# Information-dependent enrichment analysis reveals time-dependent transcriptional regulation of the estrogen pathway of toxicity

**DOI:** 10.1007/s00204-016-1824-6

**Published:** 2016-09-03

**Authors:** Salil N. Pendse, Alexandra Maertens, Michael Rosenberg, Dipanwita Roy, Rick A. Fasani, Marguerite M. Vantangoli, Samantha J. Madnick, Kim Boekelheide, Albert J. Fornace, Shelly-Ann Odwin, James D. Yager, Thomas Hartung, Melvin E. Andersen, Patrick D. McMullen

**Affiliations:** 1 The Hamner Institutes for Health Sciences, Research Triangle Park, NC USA; 2 Center for Alternatives to Animal Testing (CAAT), Department of Environmental Health Sciences, Bloomberg School of Public Health, Johns Hopkins University, Baltimore, MD USA; 3 Agilent Technologies, Inc., Santa Clara, CA USA; 4 Department of Pathology and Laboratory Medicine, Brown University, Providence, RI USA; 5 Department of Biochemistry and Molecular and Cellular Biology, and Lombardi Comprehensive Cancer Center, Georgetown University, Washington, DC USA; 6 Department of Environmental Health Sciences, Bloomberg School of Public Health, Johns Hopkins University, Baltimore, MD USA; 7 Center for Alternatives to Animal Testing-Europe, University of Konstanz, Constance, Germany; 8 ScitoVation, LLC, 6 Davis Drive, PO Box 110566, Research Triangle Park, NC 27709 USA

**Keywords:** Endocrine Disruption, Bioinformatics, Gene Expression, Enrichment Analysis, Pathways of Toxicity

## Abstract

The twenty-first century vision for toxicology involves a transition away from high-dose animal studies to in vitro and computational models (NRC in Toxicity testing in the 21st century: a vision and a strategy, The National Academies Press, Washington, DC, 2007). This transition requires mapping pathways of toxicity by understanding how in vitro systems respond to chemical perturbation. Uncovering transcription factors/signaling networks responsible for gene expression patterns is essential for defining pathways of toxicity, and ultimately, for determining the chemical modes of action through which a toxicant acts. Traditionally, transcription factor identification is achieved via chromatin immunoprecipitation studies and summarized by calculating which transcription factors are statistically associated with up- and downregulated genes. These lists are commonly determined via statistical or fold-change cutoffs, a procedure that is sensitive to statistical power and may not be as useful for determining transcription factor associations. To move away from an arbitrary statistical or fold-change-based cutoff, we developed, in the context of the Mapping the Human Toxome project, an enrichment paradigm called information-dependent enrichment analysis (IDEA) to guide identification of the transcription factor network. We used a test case of activation in MCF-7 cells by 17β estradiol (E2). Using this new approach, we established a time course for transcriptional and functional responses to E2. ERα and ERβ were associated with short-term transcriptional changes in response to E2. Sustained exposure led to recruitment of additional transcription factors and alteration of cell cycle machinery. TFAP2C and SOX2 were the transcription factors most highly correlated with dose. E2F7, E2F1, and Foxm1, which are involved in cell proliferation, were enriched only at 24 h. IDEA should be useful for identifying candidate pathways of toxicity. IDEA outperforms gene set enrichment analysis (GSEA) and provides similar results to weighted gene correlation network analysis, a platform that helps to identify genes not annotated to pathways.

**Electronic supplementary material:**

The online version of this article (doi:10.1007/s00204-016-1824-6) contains supplementary material, which is available to authorized users.

## Introduction

Much of what we understand about the effects of toxic compounds on human health comes from decades of experiments with animals. This knowledge currently underwrites many of the safety regulations concerning exposures to hazardous compounds in commercial, industrial, and environmental applications. The testing strategies for these in-life animal tests are expensive, time consuming, and exorbitant in the use of animals (Cooper et al. 2006; Hartung and Rovida 2009). Differences between human biology and laboratory animals confound assessing human safety from exposure to a compound with animal studies (Hartung 2009). Additionally, extrapolating from high-dose conditions typically required for in vivo animal testing to chronic exposures relevant to human safety is problematic because of nonlinear dose–response relationships at high treatment levels. Together, these facts argue for new approaches in toxicity testing based on human biology (NRC 2007; Andersen and Krewski 2009, 2010).

The development of in vitro toxicity assays and computational models may be able to replace traditional in-life animal testing. High-throughput in vitro screening batteries designed to assess mode of action and hazard are currently being used to prioritize compounds for conventional in-life testing (i.e., the EPA ToxCast and NIEHS Tox21 programs). Integrating prior knowledge about biological pathways with data from screening programs yields models that are predictive of dose–response behaviors from in vivo testing results (Thomas et al. 2013a; Rotroff et al. 2014). However, these approaches rely heavily on knowledge of the underlying pathway of toxicity (PoT)—the mechanism by which exposure to a toxicant leads to an adverse biological outcome (Kleensang et al. 2014; Hartung and McBride 2011). For many commercially important chemicals, their PoTs are poorly understood. As we move forward with new in vitro technologies, it would be valuable to develop tools for deriving PoT de novo. With this goal, the Human Toxome project (Bouhifd et al. 2014) was started in an effort to employ—omics technologies to start a catalogue of PoTs.

In vivo, dose–response relationships of short-term full-genome gene expression experiments are consistent with those from phenotypic endpoints in 2-year bioassays (Thomas et al. 2013b). This observation indicates that microarray and other high-throughput experiments might also help define PoTs without having to rely on incomplete and possibly misleading literature on phenotypic response (Ioannidis 2005; Hartung 2013). To demonstrate the value of this strategy, we examined estrogen receptor signaling in the MCF-7 human breast cancer cell line as a model of estrogenic signaling.

Exposure to exogenous estrogens has been linked to deleterious reproductive and developmental effects and breast and uterine cancers. Estrogens act by binding to various estrogen receptors, including ERβ, GPER, and various ERα isoforms, i.e., ERα36 and ERα46 (Barkhem et al. 1998; Flouriot et al. 2000; Maggiolini et al. 2004; Wang et al. 2006). Working in concert, these receptors orchestrate estrogen-dependent processes through regulation of transcriptional programs in various tissues. However, comparison between gene expression datasets and high-throughput chromatin immunoprecipitation sequencing (ChIP) has revealed a relatively small overlap, suggesting that cis-activation through ERs is an inadequate description of the ER network. These findings indicate that there are additional aspects that need to be considered to connect the molecular initiating event (estrogen binding to its various receptors) to an adverse cellular outcome (defined here as altered proliferation).

In addition to estrogens acting directly through ERα and ERβ, there is increasing evidence for regulatory contributions from additional transcription factors (O’Lone et al. 2004). ERα interacts with a number of transcriptional modulators, including AP-1 (Zhao et al. 2010), Sp1 (Schultz et al. 2003), SNCG (Jiang et al. 2003), and Sin3A (Ellison-Zelski et al. 2009). Non-genomic signaling, originating from estrogens binding to the G-protein-coupled receptor GPER or from ERα isoforms anchored to the plasma membrane, initiates kinase cascades that drive transcription through mechanisms not associated with receptors binding to estrogen receptor response elements.

Predicting the transcription factors responsible for a cellular response would significantly contribute to PoT identification (Essaghir et al. 2010; Shen et al. 2011; Maertens et al. 2015). However, traditional approaches for identifying transcription factors from gene expression patterns use data from a small subset of the genome. Here, we developed a novel approach to investigate the transcription factor network responsible for estrogen-mediated transcriptional changes that makes use of a higher proportion of the biological information than conventional methods. We first performed gene expression microarray experiments by exposing MCF-7 breast cancer cells to the native estrogen, 17β-estradiol (E2). By combining the observed gene expression changes with publically available ChIP data, we generated a putative gene-regulatory network.

## Methods

### Cell culture

MCF-7 cells were purchased from the American Type Culture Collection (ATCC, Manassas, VA, USA, No. HTB-22, lot number 5938874). MCF-7 stock cells tested negative for 40 Mollicutes species mycoplasma contaminations with the GRCF’s mycoplasma test that uses a PCR based MycoDtect™ kit from Greiner Bio–One North America, Inc. (Monroe, NC) to PCR amplify the 16S–23S intergenic spacer region with a highly conserved fluorescent primer pair. Cells were seeded at a density of 300,000 cells/well in six-well plates and allowed to grow for 72 h in complete growth media composed of DMEM-F12 (GIBCO, Life Technologies, Grand Island, NY, USA, No. 11309) supplemented with 10 % fetal bovine serum (Atlanta Biologicals, Norcross, GA, USA, No. S11150), non-essential amino acids (GIBCO, Life Technologies, No. 11140), 10 μg/mL bovine insulin (Akron Biotech, Boca Raton, FL, USA, No. AK8213), and gentamicin (Invitrogen, Life Technologies, No. 15710) in bisphenol-A-free culture flasks. After 72 h, cells were rinsed with PBS and placed in treatment media composed of DMEM/F12 supplemented with 5 % dextran charcoal-stripped fetal bovine serum (DCC, Gemini Bio-products, Sacramento, CA, US, No. 100-119), non-essential amino acids, 6 ng/mL bovine insulin, and gentamicin (Invitrogen 15710064 as per protocol) for 48 h. Cells were then exposed to 17β estradiol (E2, Sigma-Aldrich, St. Louis, MO, USA, No. E8875) or vehicle control dimethylsulfoxide (DMSO, Sigma Aldrich, No. D8418) in fresh treatment media for 2, 4, 8, and 24 h. Samples were scraped into TRI Reagent (Sigma-Aldrich, No. T9424) and stored at −80 °C until RNA isolation and q-PCR analysis.

### Gene expression microarray experiments

Total RNA from MCF-7 cells was extracted using TRIzol Reagent (Thermofisher No. 10296028) according to manufacturer’s instruction, and purified using RNeasy Mini Kit (Qiagen). Purified RNA was quantified by using NanoDrop ND-1000 spectrophotometer and the quality of RNA was analyzed by using Agilent Bioanalyzer (Agilent). In total, 100 ng of total RNA from treated and control cells was converted into cDNA and then into labeled cRNA using Agilent Low Input Quick Amp Labeling Kit (Agilent). The resulting cRNA was labeled with Cy3. Labeled cRNAs were then purified, and RNA concentration and dye incorporation were measured using NanoDrop ND-100 (Thermo Scientific ND-2000) spectrophotometer. Hybridization to Agilent SurePrint G3 human whole-genome 8 × 60 K microarray (Agilent) was conducted following the manufacturer’s protocol. Microarrays were scanned with an Agilent DNA microarray scanner. Feature Extraction (11.5.1.1 version, Agilent) was used to filter, normalize, and calculate the signal intensity and ratios. Processed data were subjected to GeneSpring (Agilent) analysis. In order to ensure sufficient statistical power, microarray experiments were performed in triplicate. Gene expression data are available via the NCBI Gene Expression Omnibus (GSE84981).

### Gene expression analysis

Data from microarray experiments were analyzed using GeneSpring (Agilent) software. Raw data were imported and quantile-normalized. Fold-change expressions for the probes were calculated by calculating the ratio of change from probes in time-matched controls. Significance for the change was computed using a *t* test and corrected for multiple tests using FDR correction. Genes were then assigned to their respective probes using the annotation files created by Agilent for the microarray plates used. No fold-change cutoff was applied to gene expression results.

### Transcription factor database curation

Several compendia exist of transcription factor–target interaction that we can use to uncover the regulatory network through which estrogen acts. For this study, we used ChIP-X enrichment analysis (ChEA) database (Kou et al. 2013) and the Encode database (Bernstein et al. 2012). The databases were combined to increase coverage of either one of them. This combined database was used to calculate enrichment using IDEA and gene set enrichment analysis (GSEA).

### Information-dependent enrichment analysis (IDEA)

**Fig. 1:**
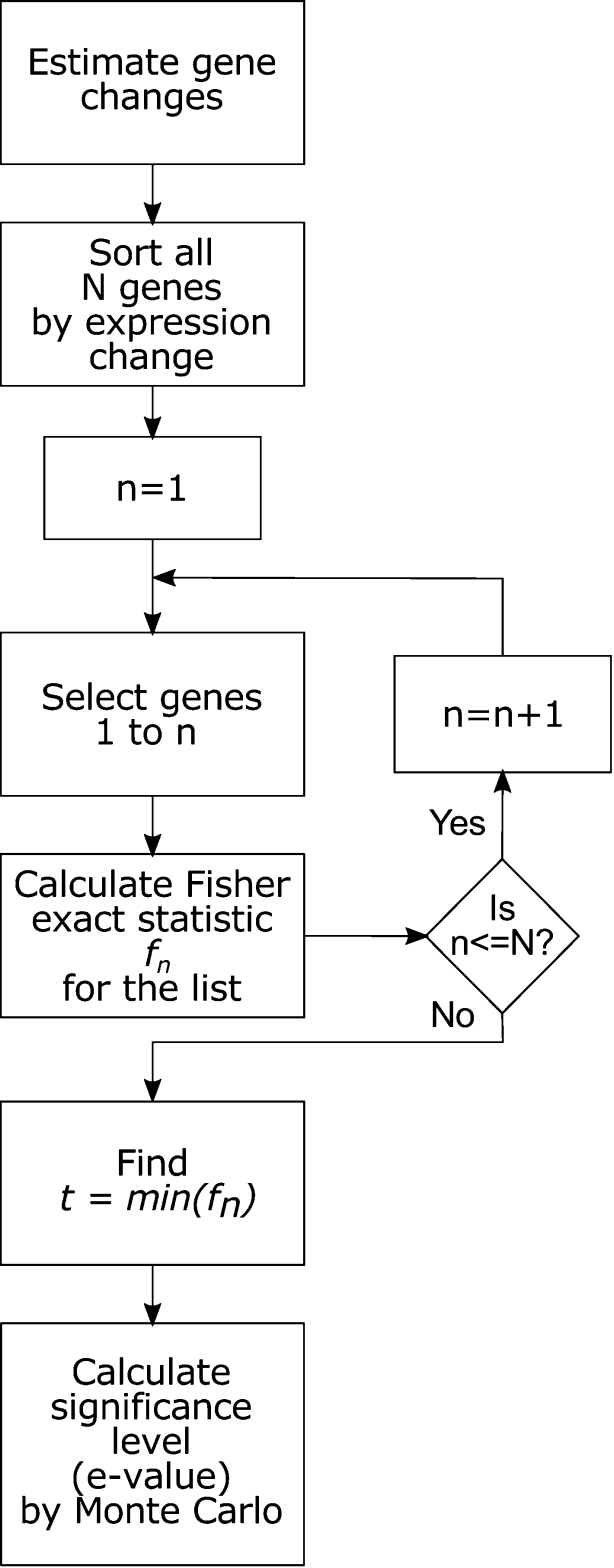
Flowchart for computing enrichment using the IDEA algorithm. Genes are split into up and downregulated sets and each set is ordered based on absolute fold-change of expression. An increasingly larger set of genes is selected for calculating enrichment of ontology categories using Fisher’s exact test. The most significant *p* value achieved through this process is compared against *p* values obtained for randomized sets of genes to calculate the final *e* value. The *flowchart* represents the process that is repeated for calculating enrichment for each category from the database

Our approach calculates over-representation across multiple sets of upregulated or downregulated genes in search of the group of genes that yields the most significant enrichment. The workflow for calculating enrichment using microarray data and transcription factor database with IDEA is represented in Fig. 1. Consider the set of *N* genes identified as upregulated. Let *δ*_*i*_ describe the relationship between a transcription factor and gene *i*, where 0 ≤ *i* ≤ *N* and *δ*_*i*_ = 1 if the transcription factor regulates gene *i*. From the set of all upregulated genes, a set {*E*_*n*_} of the *n* most highly upregulated genes can be defined. This sample of *n* genes contains ∑_{*i*<*n*}_ *δ*_*i*_ genes regulated by the transcription factor. The enrichment probability *f*_*n*_ of the association between a transcription factor and the set of genes is calculated directly using a Fisher’s exact test. Specifically, *f*_*n*_ reflects the probability that a gene selected from {*E*_*n*_} and a gene selected from the genome at large have equivalent likelihood of being regulated by the transcription factor. *f*_*n*_ can be calculated for all values of *n* and test statistic *t* defined as the min_*n*_ *f*_*n*_.

To determine whether the transcription factor is associated with the set of *N* genes, we used Monte Carlo hypothesis testing. A distribution {*t* ^0^} of null-model test statistics was established by permuting the *N* genes and calculating $f_{n}^{0}$ for each permutation (Supplemental Fig. S1). The best estimate of the probability *e* that *t* is consistent with the null model is determined by quantile function of {*t* ^0^} (Supplemental Fig. S2). These results were obtained using a distribution of 1000 null-model test statistics.

This procedure was repeated for all transcription factors in the database and for both up- and downregulated gene sets. The Benjamini–Hochberg multiple test correction was applied to the *e* values. The same procedure was repeated with KEGG and Reactome ontologies to establish effects of estrogen exposure on biological processes. For a practical example of IDEA calculations using a prototype data set, see Supplementary Methods.

### Weighted gene correlation network analysis (WGCNA)

A signed weighted gene correlation network analysis (WGCNA) network (Langfelder and Horvath 2008) was generated on the 7000 most highly expressed genes at 8 h as determined by rank means expression. The network was derived based on a signed Spearman correlation using a β of 8, and clustered into modules using dynamic tree cut with a height of 0.25 and a deep split level of 3, and a reassign threshold of 2. The eigenmodules—essentially the first principal component of the modules—were then correlated with dose. Each module that had a statistically significant correlation with dose was analyzed for transcription factors using the ChEA 2015 dataset accessed via EnrichR (Kou et al. 2013) restricted to MCF-7 cells.

## Results and discussion

### Response of MCF-7 cells to estrogen

The gene expression response of MCF-7 breast carcinoma cell line to 17β-estradiol (E2) has been extensively documented (O’Lone et al. 2004). However, the studies in the MCF-7 experimental system (Rae et al. 2005; Carroll et al. 2006; Chang et al. 2006; Creighton et al. 2006; Fan et al. 2006; Frasor et al. 2006; Gaube et al. 2007; Kininis et al. 2007; Lin et al. 2007a, b; Bourdeau et al. 2008; Chang et al. 2008) are inconsistent when analyzed at the gene level (Ochsner et al. 2009; Jagannathan and Robinson-Rechavi 2011) due to subtle differences in cell culture and treatment conditions. Because none of the extant studies includes a comprehensive concentration and time response, and to ensure we could evaluate effects of dose and time without combining disparate studies, we performed a comprehensive dose and time response transcriptomic study performing gene expression microarray analysis on MCF-7 cells treated with 0.01, 0.1, and 1 nM E2 for 2, 4, 8, and 24 h.

**Fig. 2:**
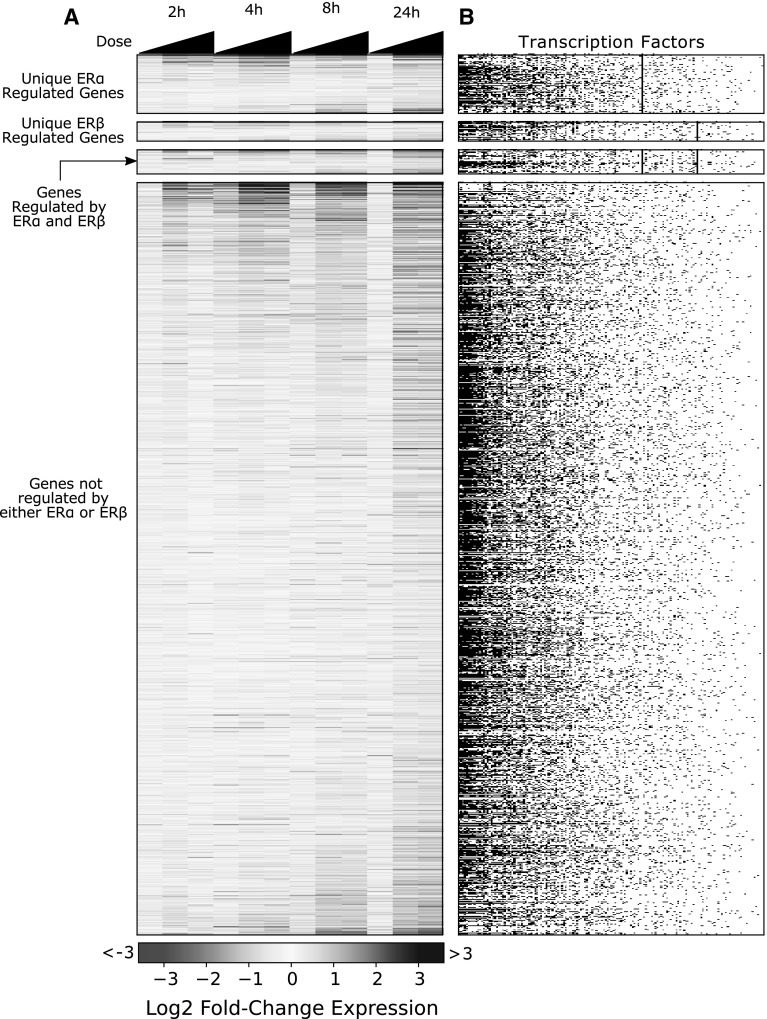
Genomic response of E2-treated MCF-7 cells. **a** Matrix of expression changes for all differentially expressed genes in response to E2 treatment. *Each row* indicates a gene; *each column* indicates a treatment condition increasing in dose (0.01, 0.1, and 1 nM) from *left* to *right*. Genes were categorized as being regulated by ERα and/or ERβ if transcription factor binding was documented for respective transcription factors in the combined ChEA and encode database. A small fraction of the differentially expressed genes have been shown to bind ERα and/or ERβ in previous studies. **b** Transcription factor regulation matrix. *Each row* is a gene and each column is a transcription factor. *Black dots* indicate that gene is shown to have the corresponding transcription factor binding

The number of genes identified as differentially expressed using traditional statistical analysis (FDR-corrected *p* values <0.05) in cells treated with 1 nM E2 varied substantially with time and concentration; from zero genes after 2-h exposure to 4113 genes after 24 h (Fig. 2; Table 1). This increase in number of differentially expressed genes is not monotonic, with 547 genes identified at 4 h and only four genes identified at 8 h post-treatment. Because the identification of differentially expressed genes depends on experimental factors that drive statistical power, it is unclear whether this decrease in differentially expressed genes with time is biologically meaningful. This observation is common to functional genomics experiments that test thousands of hypotheses in parallel. In our case, as we try to reconstruct the transcription factor network, the inability to determine whether these results are biological or an analytical artifact of statistical methods poses a challenge for correctly interpreting data.

**Table 1:** Number of genes differentially expressed following E2 treatment in MCF-7 cells (FDR *p* value ≤0.05)

	2 h		4 h		8 h		24 h	
	Up	Down	Up	Down	Up	Down	Up	Down
0.01 nM	3	9	48	39	14	14	0	0
0.1 nM	205	120	670	312	229	180	1350	954
1.0 nM	0	0	375	172	3	1	2101	2012

Because the lists of differentially expressed genes derived from microarray experiments are determined by statistical power (e.g., number of replicates, RNA isolation protocol, and microarray platform) as well as biology, using standard over-representation analysis to assign functional ontology descriptions to sets of genes is likely be misleading. Classical overrepresentation analysis suffers from a number of shortcomings. Gene expression changes are aggregated into lists of up- and downregulated genes based on statistical significance, magnitude of change, or some combination of these and other factors. These choices result in a list of genes with the most extreme response. However, it is rare that a gene encodes a protein that is solely responsible for the cellular response to stimuli. In most cases, multiple genes are transcribed to varying degrees to bring about a cellular change. Using an arbitrary analytical threshold cutoff (i.e., *p* value or fold change) does not take into account the diverse nature of gene transcription in relation to PoTs. This traditional approach is unlikely to capture subtle effects at low doses or early time points where very few genes are identified as significantly differentially expressed. Indeed, this approach often results in no categories enriched at low exposures and many non-informative categories enriched at high exposures. Additionally, because statistical cutoffs are not biologically motivated and the number of statistically significant genes may be different for each experimental condition, it is difficult to compare relative enrichments for categories across experimental conditions.

To address this problem, we developed an alternative approach for assessing enrichment from high-throughput data. Our approach has the advantage of being relatively insensitive to variability in statistical power in assignment of differential expression and makes use of a greater portion of the gene expression data to determine enrichment for transcription factor binding or functional ontology.

### The information-dependent enrichment analysis (IDEA) algorithm for calculating enrichment

IDEA bypasses the limitations of existing methods by avoiding differentially expressed gene lists and instead uses the entire set of microarray data to create a gene list ordered by expression values. This approach avoids attempts to balance the sensitivity and specificity with statistical cutoffs and concerns about bias in the background.

**Fig. 3:**
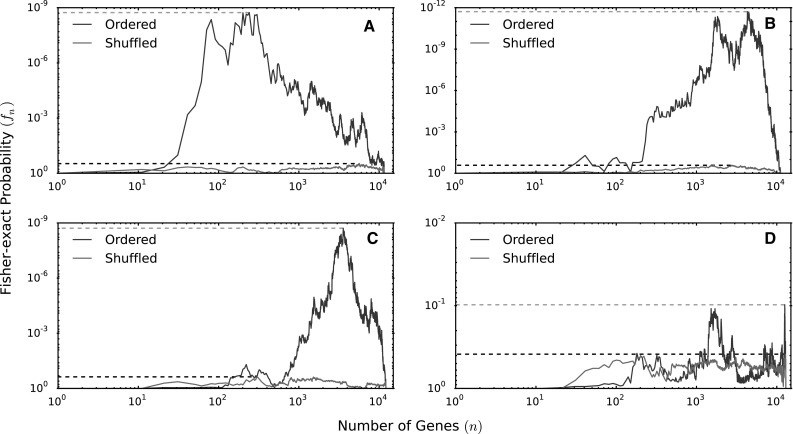
Enrichment of ERα binding for genes up- and downregulated by E2 in MCF7 cells. The enrichment (i.e., Fisher’s exact probability, *blue traces*) varies considerably depending on the number of genes used for the calculation for **a** genes upregulated 24 h post-treatment, **b** downregulated 24 h post-treatment, **c** upregulated 2 h post-treatment and **d** downregulated 2 h post-treatment. Enrichment for genes ordered by decreasing fold-change (*blue*) and shuffled sets of background genes (*green*) are shown. *Dashed lines* indicate lowest enrichment *p* value achieved by a subset of the gene list sorted by decreasing absolute fold change (*red*) or randomly shuffled (*black*). Once the gene lists are sorted by fold change the lowest enrichment *p* value changes by 9 orders of magnitude (**a**–**c**). For genes downregulated at 24 h, **d** sorting the list by fold change does not improve the *p* value of enrichment. This indicates an inherent lack of information about ERα enrichment in the gene expression pattern in genes downregulated at 24 h (color figure online)

The IDEA algorithm calculates over-representation across multiple sets of upregulated or downregulated genes in search of the group of genes that yields the most significant enrichment (see “Methods” section, Fig. 1, Supplementary Methods for a rigorous description of the algorithm). The statistical power of over-representation calculations varies considerably depending on the number of genes used for the calculation (Fig. 3). A static set of genes—such as that produced by traditional gene expression analysis—may not yield optimal enrichment. Alternatively, considering gene sets of different sizes allows one to identify the genes that provide the maximum information about enrichment. Effectively, this allows identification of enrichment patterns from relatively weak transcriptional changes, such as those produced by exposures to low chemical concentrations for short durations.

IDEA identifies enrichment patterns in expression signatures by sorting genes based on their response to experimental perturbation. This sorting uses the magnitude of expression change and does not depend on arbitrary pre-defined thresholds (statistical or otherwise) of change. If there is a coherent enrichment signature driving expression changes, one expects it to be identifiable from the most highly up- or downregulated genes (Fig. 3). The combination of fold-change-based sorting and enrichment using incrementally larger list of genes enables IDEA identify enrichment patterns that might have been missed using traditional approaches.

IDEA provides a natural basis for determining statistical significance for enrichment calculations. For each statistical test, a battery of background sets is prepared from the ensemble of all genes up- or downregulated in response to treatment. For each background set in this battery, the maximal enrichment is calculated, yielding a null-model test statistic. This collection of null-model test statistics collectively provides a null model for determining enrichment significance in the test data (see “Methods” section). Along with the actual *p* value of enrichment, the number of genes required to achieve that value provides an additional metric for comprehending cellular response. A category that requires a larger proportion of background genes to achieve peak enrichment is driven by a larger cohort of weakly responding genes, whereas if peak enrichment is achieved using a smaller number of genes, the category is being driven by a small highly responsive set.

Taking into account all the information provided by the algorithm, we can predict how the cell responds to stimuli in a time- and dose-dependent manner. We calculated enrichment for all transcription factors from our knowledgebase using IDEA. To better understand the changes in enrichment across multiple transcription factors, we created an online interactive visualization tool for exploration of our results (http://www.scitovation.com/MCF7_IDEA_Applet).

### Estrogen receptors drive the short-term transcriptional response to E2

We surveyed existing ChIP datasets to evaluate the factors driving estrogen-mediated transcriptional changes. Interestingly, ERα and ERβ—two primary estrogen transcription factors—bind only about 11 % of the differentially expressed genes. This low degree of overlap between receptor binding and gene expression regulation is in sharp contrast to other nuclear receptors that have been shown to account for approximately half of the affected genes’ expression (van der Meer et al. 2010; McMullen et al. 2014).

At 2 h, of the upregulated genes, 28 % are required to reach peak ERα and ERβ enrichment. At 24 h, this ratio shifts to only 1.7 % for ERα and 7.8 % for ERβ. In other words, the ERα and ERβ signal increases over time—at 2 h it is barely above background level, while at 24 h the signal is more distinct.

**Fig. 4:**
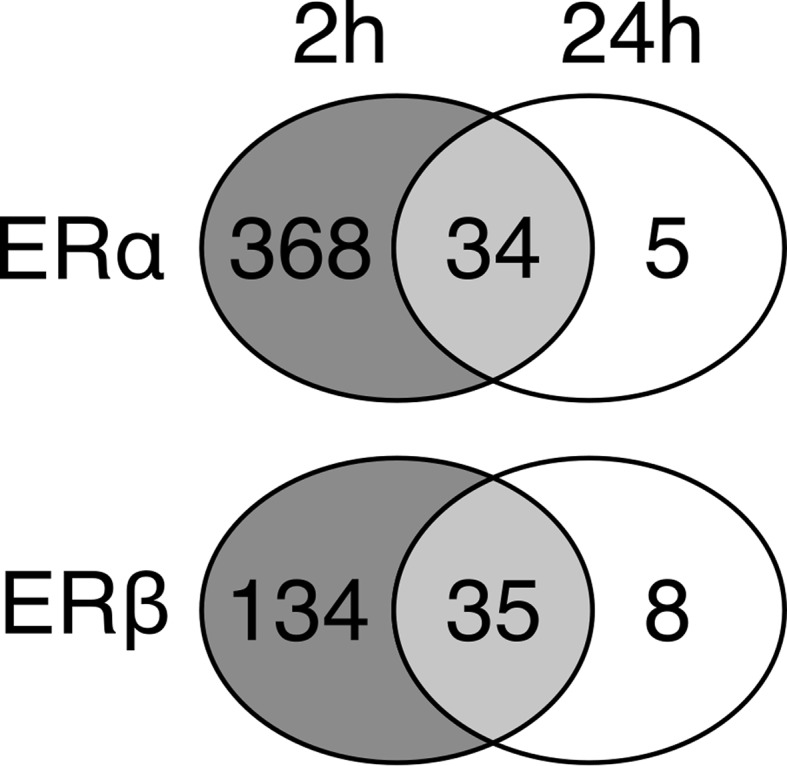
Upregulated ERα- and ERβ-associated genes are consistent over time. Overlap of genes upregulated in response to 2 and 24 h exposures of 1 nM E2 in MCF7 cells. Almost all genes contributing to enrichment of ERα and ERβ at 24 h also contributed to the enrichment at 2 h, indicating the consistency of estrogen response. While the number of ERα- and ERβ-associated genes decreases between 2 and 24 h, these genes remain highly upregulated (Fig. 2, Supplementary Fig. S3)

We investigated the large variation in number of genes needed for peak enrichment to determine whether this factor contained information about the biology of the system. Similar sets of ERα and ERβ genes are upregulated at 2 and 24 h (Fig. 4). Also, the highest responding genes at 24 h are also upregulated at 2 h. Hence, the time course of estrogen receptor enrichment shows a shift from a large suite of ER genes expressed at low level to a small set of highly expressed ER genes.

**Fig. 5:**
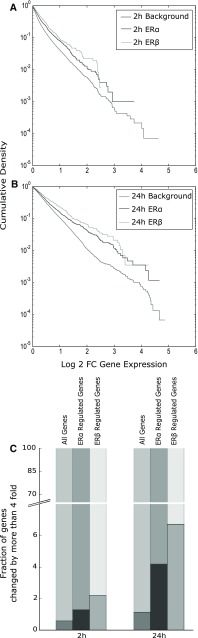
ERα- and ERβ-regulated genes are highly upregulated in response to E2 treatment. Cumulative distributions of expression changes in all upregulated genes and those that are regulated by ERα and ERβ at **a** 2 h and **b** 24 h. At 2 h, the cumulative distribution of both ERα and ERβ upregulated genes follows the background distribution closely. However, at 24 h, the distribution of genes regulated by the transcription factors deviates from substantially from background. **c** The fraction of genes in each of these treatment groups that is more than fourfold upregulated confirms that estrogen receptor-mediated genes are highly upregulated by E2 treatment. After 2 h of treatment, <0.5 % of all upregulated genes are more than fourfold upregulated. In contrast, 1.5 % of ERα-regulated and 2 % of ERβ regulated genes are changed fourfold. At 24 h, this ratio increases to 1 % when all upregulated genes are considered. However, at 24 h, substantially more ERα and ERβ regulated genes (4.2 and 6.7 %, respectively) are upregulated at least fourfold

Plotting the distribution of expression for all ERα- and ERβ-regulated genes provides further evidence to the switch in pattern of regulation (Fig. 5). At 24 h, there is a larger set of highly upregulated genes (greater than fourfold change) than in background. Alternatively, at 2 h, all the ERα- and ERβ-regulated genes track background levels of gene expression more closely. All these observations taken together indicate that at ERα and ERβ are strong drivers of the underlying expression pattern at 24 h, but their signal is mediated by only a limited subset of the full group of differentially expressed genes.

With respect to defining a signature of toxicity, this approach allows quantifying the strength of the signal at different time points. Standard overrepresentation analyses are unable to capture the gradual increase in strength of the signal. This results in misleading observations regarding the threshold like behavior of transcriptional factor signaling cascades. Using IDEA allows us to establish that a large subset of weakly upregulated or downregulated genes can indeed drive the signaling networks.

A very different pattern of enrichment is observed in the case of downregulated genes. Looking at gene expression at 2 h, there was no enrichment in ERα and ERβ profiles detected. We observed enrichment for a few other transcription factors, including ZNF217, indicating that the absence of enrichment is due to lack of ER signaling in these genes rather than a lack of gene expression. At later times, estrogen receptors are highly enriched by downregulated genes. However, the fact that their peak enrichment never requires <37 % of all downregulated genes indicates that ERα and ERβ are not key mediators of downregulated gene response.

### Growth factors drive the long term transcriptional response

Persistent exposure of MCF-7 cells to E2 induces cell proliferation (Soto and Sonnenschein 1985). We believe the shift from low-level expression of large numbers of ER-mediated genes to high-level expression of fewer ER-mediated genes results from a shift from estrogen-specific signaling to more generic cell cycle signaling. At 24-h E2F7, E2F1 and Foxm1 are enriched in addition to the ERs. Both Foxm1 and E2F1 are transcriptional activators involved in cell proliferation (Stender et al. 2007; Real et al. 2011). E2F7 represses the activity of E2F1 by binding to E2F1-responsive genes (De Bruin et al. 2003; Liu et al. 2013). However, at 24 h, E2F7 is only enriched in the set of upregulated genes. Since it is not enriched in downregulated genes, E2F7 enrichment is not inhibiting the generic cell cycle signature promoted by E2F1.

ZNF217, a transcription factor that has been implicated in cell division and differentiation in many cancers (Zhu et al. 2005; Littlepage et al. 2012; Rahman et al. 2012), is enriched by downregulated genes at all time points. High levels of ZNF217 mRNA are a marker of poor prognosis in breast cancer (Littlepage et al. 2012). ZNF217 primarily acts by repressing genes that halt cell cycle, thereby promoting cell growth and differentiation (Thollet et al. 2010). This loss of gene expression is consistent with the role of ZNF217 as a repressor that is essential to proliferation in breast cancer cells (Thollet et al. 2010).

### Relationship to gene set enrichment analysis (GSEA)

GSEA (Subramanian et al. 2005) attempts to use a priori gene set information to calculate enrichment of gene lists. We applied the GSEA algorithm to our combined transcription factor database to better understand the relationship between IDEA and existing methods (Subramanian et al. 2005). The results agree closely with those obtained by us. At 2 h, only ERα, ERβ, and NOTCH1 are enriched in cells treated with 1 nM E2 using recommended parameters at a FDR of <25 %, the value used by the creators of GSEA as a valid cutoff for establishing enrichment (Subramanian et al. 2005). At 24 h, 69 transcription factors are identified as enriched in treated cells. Additionally, at 2 h, ERα and ERβ are highly enriched for treated cells, whereas at 24 h the E2F family of proteins is highly enriched for the treated cells. This result aligns with our hypothesis that a proximate ER network feeds into the generic cell cycle processes to effect proliferation and other phenotypic alterations associated with E2 treatment.

GSEA discerns differences in enrichment between two experimental conditions (often a treatment and a control) by attributing enrichment of each gene set to one of the two conditions. When using GSEA to compare enrichment between estrogen-treated and untreated cells, transcription factors associated with downregulated genes and those that have no effect are both identified as enriched in untreated cells. As shown above, ERα and ERβ are associated with large sets of both upregulated and downregulated genes. The similarities and differences in the composition of these gene sets and their expression patterns are essential in uncovering the underlying transcription factor network. At longer exposures, GSEA identifies enrichment in ERα and ERβ in untreated cells but ignores the small set of highly upregulated genes driven by these transcription factors. This rigidity inherent to the GSEA method hinders its utility in interpreting results, whereas the same transcription factors may be responsible for both activation and repression of genes through different pathways. Finally, the results of GSEA are dependent on the choice of weight function for calculating the enrichment statistic, whereas IDEA relies on the statistics of the hypergeometric distribution to calculate enrichment and places no a priori conditions on the possible enrichment categories.

### Evidence for cell cycle signaling from functional ontologies

Cell cycle is controlled by a large number of transcription factors. Hence it was necessary to ensure that enrichment in E2F family of proteins is indicative of global cell cycle signaling in the cell. Functional ontologies like Kyoto encyclopedia of genes and genomes (KEGG) and Reactome attempt to assign genes to functional categories based on information curated from experimental results. These databases are better at identifying processes (i.e., cell cycle, metabolism, etc.) that depend on a relatively large section of the genome to be expressed. As such they complement transcription factor databases that capture processes regulated by a small subset of genes in the genome.

**Fig. 6:**
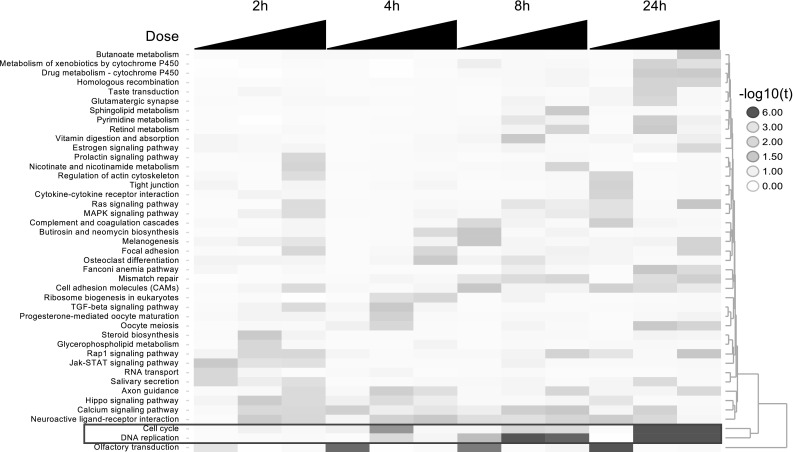
Clustering *t* values for enriched KEGG pathways. The map shows enrichment values for various KEGG pathways across E2 concentration and exposure time. *Each row* represents a KEGG category while *each column* represents a treatment condition increasing in dose (0.01, 0.1, 1 nM E2) from *left* to *right*. The enrichment values were clustered using a hierarchical clustering algorithm using Euclidian distance between decimal logarithm of *t* values as a clustering metric. Cell cycle-related pathways cluster independent of all other pathways (*green box*). Cell cycle is strongly enriched only at 24 h post-treatments, while DNA replication is enriched at both 8 and 24 h post-treatment. This is consistent with changing transcription factor enrichment observed with IDEA (color figure online)

**Fig. 7:**
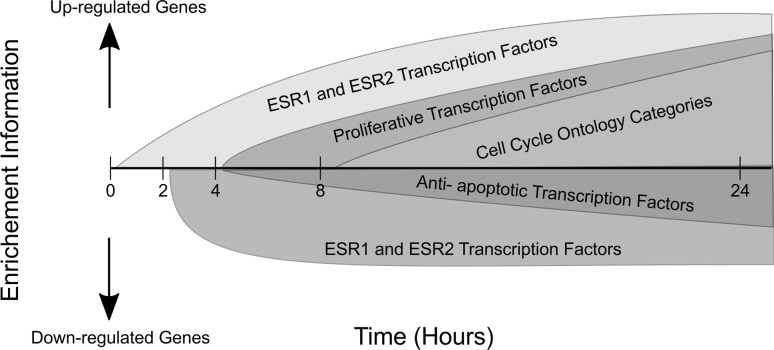
Summarizing key events in estrogen signaling. Genes upregulated by ERα and ERβ already show a strong expression pattern at 2 h post-treatment. This pattern continues to get stronger with time. Generic proliferative transcription factors including E2F1 and E2F4 are enriched at 8 h post-exposure but not at earlier times. Concordantly, genes involved in cell cycle and proliferation also show a strong upregulated pattern. Some ERα and ERβ genes are downregulated in response to E2 treatment; their expression patterns, however, remain unchanged over time

To investigate the hypothesis of altered cell cycle signal appearing only after longer exposures, we calculated the enrichment of functional categories in both Reactome and KEGG using the IDEA algorithm. We observed a very clear temporal pattern of enrichment of cell cycle-related categories. At 2 and 4 h, none of the cell cycle-related categories were enriched. However, at 8 h, we observed enrichment of some cell cycle-related categories like DNA replication. Finally, at 24 h post-treatment, all mitotic cell cycle-related categories in both Reactome and KEGG were significantly enriched. Furthermore, the numbers of genes needed for peak enrichment at 24 h were less than those needed at 8 h, indicating stronger information content in the enrichment signal at 24 h. We also clustered the enrichment profiles obtained from KEGG ontology using a hierarchical clustering algorithm with Euclidian distance metric between decimal logarithms of *t* values (Fig. 6). The clustering showed a similar response with signaling pathways being activated as early as 2 and 4 h. Cell cycle and DNA replication were only enriched at 8 and 24 h. Figure 7 illustrates the general time dependence of key transitions in transcription factor and functional ontology enrichment patterns.

### De novo network analysis of estrogen perturbation

To investigate the data from a methodology that is blind to a priori knowledge of transcription factor binding sites and is relatively insensitive to concerns about technical bias, we used weighted gene correlation network analysis (WGCNA) to build a de novo network from the data using the dose response curve at 8 h—notably a time point where only four genes were significantly expressed in response to E2. Correlation methods offer an additional alternative to using differentially expressed genes for downstream analysis, as they take advantage of a larger portion of the data and allow for the investigation of links between genes (Maertens et al. 2015). Moreover, WGCNA assigns genes to modules based on a graph theoretical algorithm and tests for significance between the modules and experimental factors (here, the dose–response curve). The added value for identifying PoT has been recognized earlier (Andersen et al. 2015; Rahnenfuhrer and Leist 2015).

Despite a relatively weak signal in terms of differentially expressed genes at that the 8-h time point, the network derived from the data contained five modules that were significantly correlated with E2 concentration. To understand the relationship between the transcriptional factors coordinating the gene expression and the modules, each module was analyzed for transcription factors using the ChEA dataset but restricted to MCF-7 cells. In addition to ESR1 and ESR2, there were also well-known estrogen response pathway transcription factors such as GATA3 and cancer-related transcription factor HIF1. Moreover, both ZNF217 and TFAP2C were identified as a significant transcription factor in each module correlated with dose and as expected several transcriptional modules coincident with the enriched transcription factors for upregulated genes identified with IDEA (Supplementary Table A, D).

While TFAP2C is neither annotated to estrogen-responsive pathways in either the KEGG estrogen signaling pathway (Kanehisa and Goto 2000) nor does have any GO annotations relating to estrogen, it is a key regulator of hormone responsiveness at multiple levels. It acts both directly, by regulating ERα transcription, and indirectly, by recruiting key estrogen pioneer transcription factors GATA3 and FOXA1, and also modulates several downstream signaling pathways (Cyr et al. 2015). In vitro TFAP2C attenuation leads to a lack of mitogenic response to estrogen and in vivo decreased hormone-responsive tumor growth of breast cancer xenografts (Woodfield et al. 2007). Clinically, higher TFAP2C scores correlate with poorer survival for breast cancer patients (Perkins et al. 2015). Moreover, both TFAP2C and ZNF217 gene expression levels were correlated with estrogen receptor status in breast cancer dataset from The Cancer Genome Atlas, indicating that the significance of these genes for in vivo biology.

SOX2 was also identified in several of the modules. One key step in the regulation of breast tumor-initiating cells takes place when ERα downregulates miR-140 (Zhang et al. 2012), leading to increased SOX2. SOX2 is considered a key regulator of stem-cell self-renewal and specifically in breast cancer tissue is thought to promote non-genomic estrogen signaling while simultaneously acting to amplify estrogen’s signal by increasing the nuclear levels of phospho-Ser118-ERα (Vazquez-Martin et al. 2013). Expression of SOX2 is increased in early stage breast tumors, over-expression of SOX2 increased mammosphere formation, and SOX2 knockdown prevented mammosphere formation in stem cells and tumor formation in a xenograft tumor initiation model (Leis et al. 2012). Both TFAP2C and SOX2 were also enriched using the IDEA algorithm.

The concordance between the IDEA algorithm (which also identified TFAP2C, ZNF217, and SOX2), and the transcription factors identified by WGCNA shows that the methods complement each other, and further investigation of expression analysis using WGCNA might help identify estrogen-responsive genes not annotated to ER pathways. Using standard over-representation analyses for KEGG and GO databases would not have uncovered these additional regulatory elements on the ER pathway like TFAP2C. This further highlights the need for approaching expression datasets with multiple integrative analytical approaches.

## Conclusions

The technologies driving modern biology produce large amounts data, often spanning the breadth of the genome. However, methods for extracting biological insight from the results of these experiments have lagged behind. New computational tools and visualization strategies are required to fully realize the potential of systems biology for revolutionizing toxicity testing and mapping PoTs. High-throughput tools often implicate large lists of genes for particular phenotypic responses. However, translating this information into biological knowledge remains a fundamental challenge. There also exists a persistent perception in modern biological research that more information automatically leads to greater knowledge. However, the quantity and complexity of high-throughput data is typically not directly translatable into advancing our understanding of biology.

Summarizing changes to transcriptional programs by associating them with existing literature and curated databases is a key modality for summarizing and understanding the results of high-throughput experiments. Here, we treated MCF-7 cells with E2 and calculated transcription factors over-represented by expressed genes. We also inferred the functional characteristics of those gene expression changes. Because existing enrichment approaches were insufficient for interpreting these changes, we developed a new method that makes more comprehensive use of the biological data.

Our tool, IDEA, provides a framework for observing patterns with gene expression studies. Toxicants with similar modes of action are expected to induce similar patterns of transcriptional change. However, changes in individual genes are typically not as robust as changes at the pathway level. Because IDEA summarizes gene expression changes into a small subset of transcription factors or ontology categories associated with the up- and/or downregulated genes, it has promise for identifying modes of action.

**Fig. 8:**
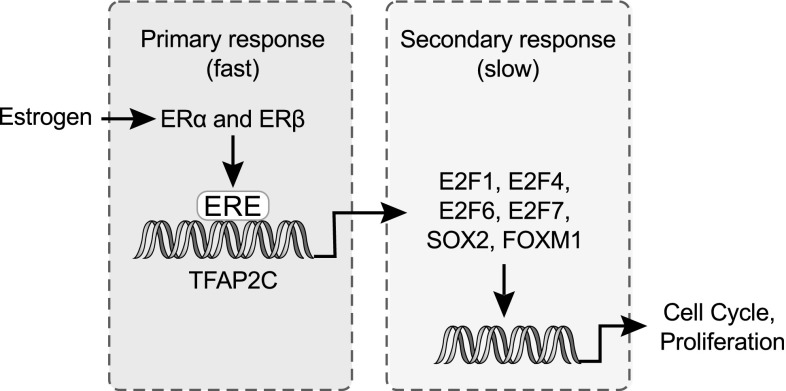
Putative transcription factor network for estrogen signaling. ERα and ERβ bind directly to DNA via estrogen response elements (EREs). This initiates transcription of a set of estrogen-responsive genes. At longer exposures, these estrogen-responsive genes initiate the transcription of a larger set of secondary transcription factors. These transcription factors then promote proliferation and suppress apoptotic genes

By considering the time course of genes regulated by various transcription factors, we found that response to estrogen involves two distinct steps (Fig. 8). During the first stage, at 2–4 h post-treatment, signaling is dominated by cis-regulation of ERα and ERβ. This primes the cells for growth. At longer exposures, only a subset of ERα- and ERβ-controlled genes is highly upregulated. Simultaneously, a large set of genes regulated by cell signaling transcription factors, including E2F1, E2F4, E2F7, and Foxm1 are upregulated. At longer exposures, cell cycle-related categories in KEGG (Kanehisa and Goto 2000) and Reactome (Fabregat et al. 2016) are enriched in upregulated genes, while apoptotic and anti-proliferative categories are enriched in downregulated genes.

We found sequential patterns of enrichment over increasing exposures by moving away from using traditional *p* value and fold-change cutoffs to define lists to be used for calculating enrichment. These cutoffs do not account for low-level, diffuse patterns of gene expression that can characterize early time points or low dose responses to exposure. Using the entire dataset rather than a limited set of highly expressed genes allowed us to investigate the cellular response at 2, 4 and 8 h post-treatment, where the number of differentially expressed genes did not yield any enrichment information regarding either transcription factor binding or cellular processes. Additionally, IDEA allowed us to obtain results under conditions where array normalization and experimental noise would have severely decreased the utility of traditional enrichment methods. IDEA captures information contained in relative expression of genes with respect to each other as opposed to an external cutoff. This feature can be used to compare enrichment results across multiple experiments providing a way to study functional enrichment results across gene expression studies originating from different labs. Along with significantly enriched pathways and transcription factors, IDEA also provides a gene list that optimizes enrichment at a given experimental condition. This gave us better insight into the strength of the signal in the data as it unfolds over both time and dose; two characteristics that can be useful for both experimental design with other bioinformatics approaches that require dimensionality reduction.

The algorithm, IDEA provides a framework for observing patterns within gene expression studies and serves as a useful a tool to investigate modes of action for multiple chemicals of the same class. It allows for calculation of enrichment at experimental conditions where very few genes are identified as being significantly expressed. The similarity of results between IDEA and WGCNA is reassuring, showing that these methods complement each other and, in combination, should provide a more nuanced characterization of estrogen PoTs. IDEA lends itself to the initial identification of candidate PoT, a process that could be followed by more targeted experiments on the path to a Human Toxome (Bouhifd et al. 2014) and a systems toxicology approach (Hartung 2013).

## Electronic supplementary material

Below is the link to the electronic supplementary material.

Supplementary material 1 (DOCX 872 kb)

Supplementary material 2 (PDF 196 kb)

Supplementary material 3 (XLSX 11 kb)

Supplementary material 4 (XLSX 144 kb)

Supplementary material 5 (XLSX 1147 kb)

Supplementary material 6 (XLSX 106 kb)
