# Supplementary material for: Information-dependent enrichment analysis reveals time-dependent transcriptional regulation of the estrogen pathway of toxicity
Source: Arch Toxicol. 2016 Sep 3;91(4):1749–62. doi: 10.1007/s00204-016-1824-6 (PMC5364265; doi:10.1007/s00204-016-1824-6)
Supplement: Supplementary file 1 — Supplementary material 1 (DOCX 872 kb) [file 204_2016_1824_MOESM1_ESM.docx]

## Supplemental Figures

Supplementary Fig. S1. Characterization of the IDEA null model. (A) Four representative simulations of *f_n_^0^* for the association of upregulated genes with ERα. Circles denote *t^0^* for each simulation. (B) Null model test statistics must be uniformly distributed in a valid Monte Carlo hypothesis test. Ensembles of *f_n_^0^* for *n*=100 (red), *n*=1000 (blue), and *n*=10,000 (green) are uniformly distributed (black dashed line) between 0 and 1.

**
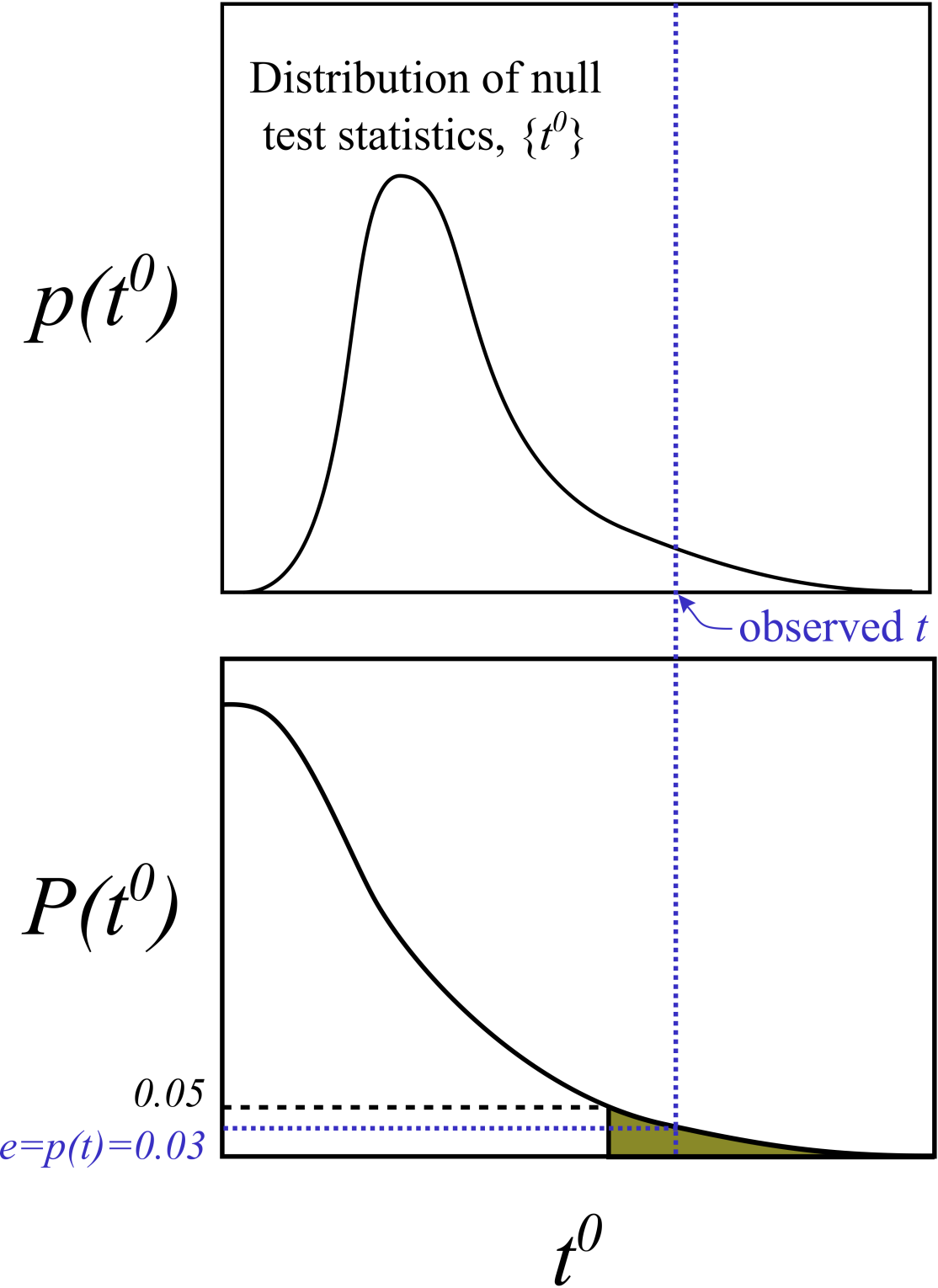
**

**Supplementary Fig. S2.** Successive simulations of *f_n_^0^* give rise to an ensemble of null model test statistics, {*t^0^*}. Significance of enrichment, *e*, is based on the relationship between the observed test statistic *t* and {*t^0^*}. The probability of observing a test statistic more extreme than *t* is the cumulative density of {*t^0^*} evaluated at *t.*


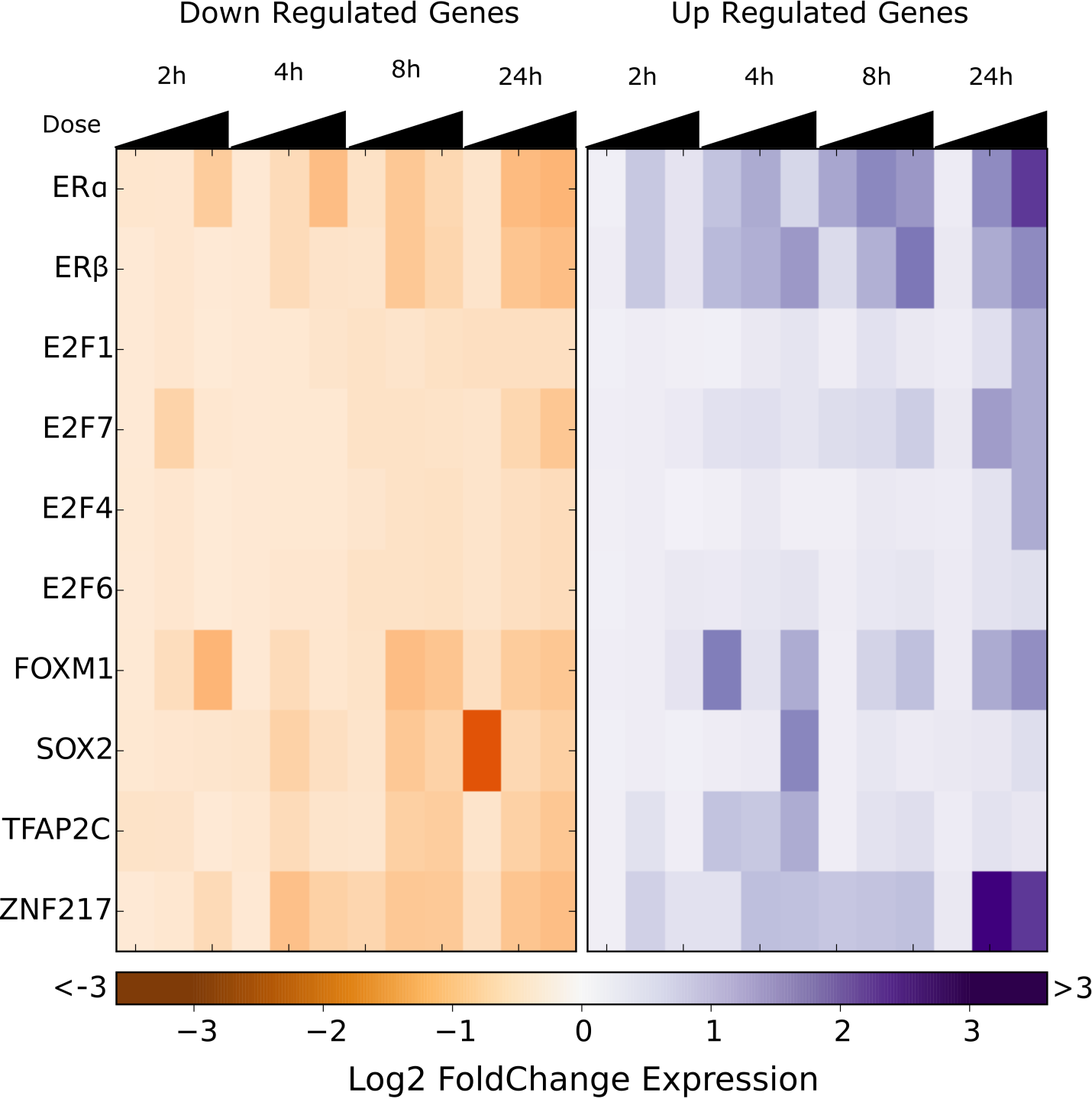


Supplementary Fig. S3. Expression for genes regulated by key transcription factors. Mean expression across all genes A) down-regulated and B) up-regulated by a given transcription factor and contributing to peak enrichment using IDEA. Mean expression for genes down-regulated by ERα and ERβ increases with increasing time. Conversely, mean expression for genes up-regulated by E2F family of transcription factors increases with time.


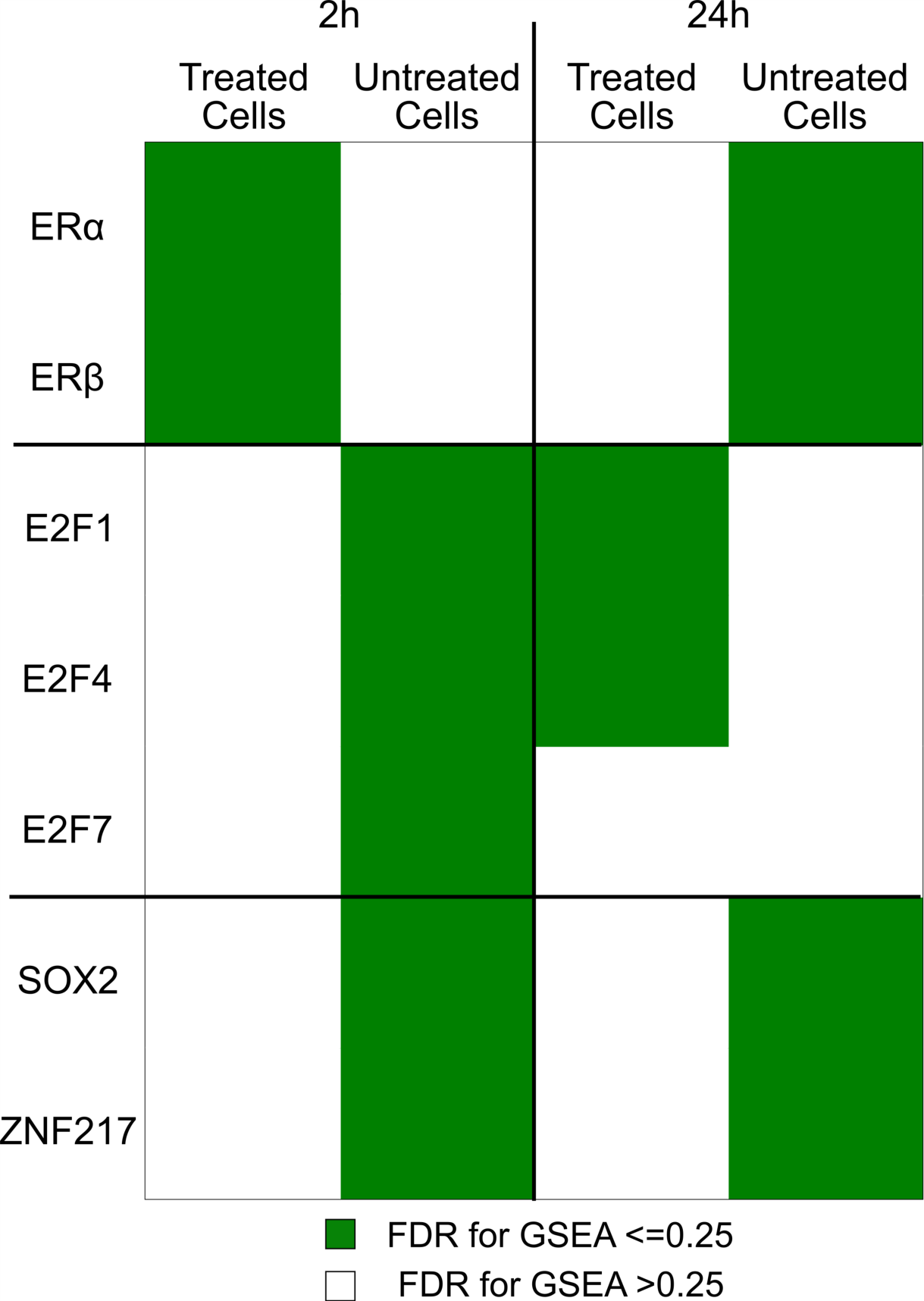


Supplementary Fig. S4 Enrichment of Key Transcription Factors using GSEA. Similar to the results obtained by IDEA, ERα and ERβ are enriched in treated cells at 2h and untreated cells at 24h, while E2F family of transcription factors shows the opposite result. It is worth noting that enrichment in untreated cells can be due to lack of any up-regulation in treated cells (as is the case with E2F family after 2h of treatment) or active downregulation in treated cells (ERα and ERβ after 24h of treatment). This difference is clearly visible with results obtained using IDEA.


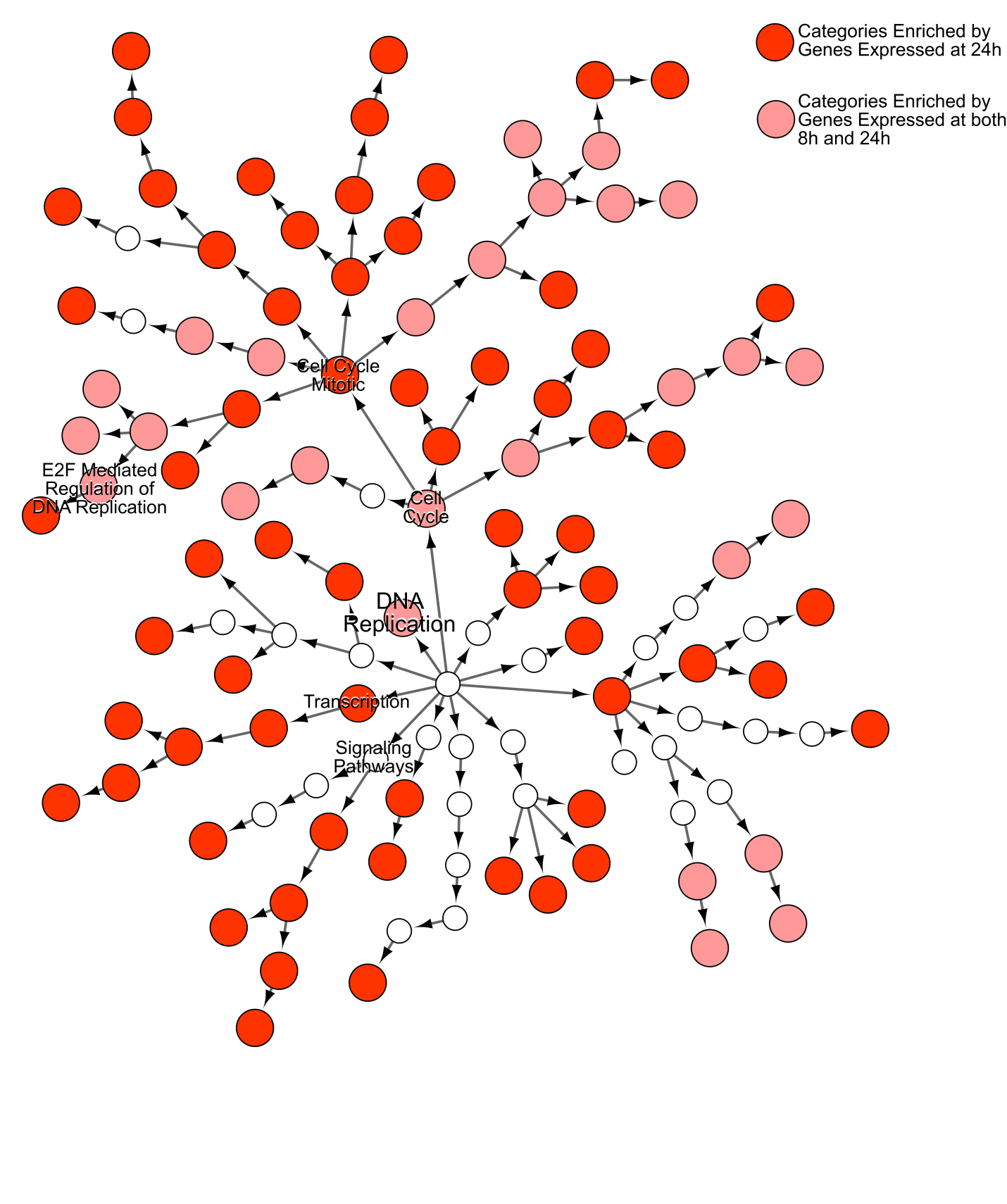


Supplementary Fig. S5. Reactome Enrichment using IDEA. Significant enrichment of cell cycle related categories is observed only at 8 and 24h post exposure.
