# Supplementary material for: Information-dependent enrichment analysis reveals time-dependent transcriptional regulation of the estrogen pathway of toxicity
Source: Arch Toxicol. 2016 Sep 3;91(4):1749–62. doi: 10.1007/s00204-016-1824-6 (PMC5364265; doi:10.1007/s00204-016-1824-6)
Supplement: Supplementary file 2 — Supplementary material 2 (PDF 196 kb) [file 204_2016_1824_MOESM2_ESM.pdf]

We start with a list of genes and their associated log fold-change over control samples.To explain the algorithm better, we will only consider gene expression data in MCF7 cells treated with 1nM E2 for 24h in this example. At the end of this algorithm, we will have calculated enrichment for all the transcription factors in our knowledgebase. The algorithm is then repeated for each treatment condition to calculate overall enrichment pattern.

Step 1 - Splitting the gene set based on direction of Regulation and sorting them on the basis of absolute fold-change

Consider a set of genes as given below

Gene Name	Expression Value
A1BG	-0.453364
A1BG-AS1	-0.47478
A1CF	0.0223632
A2M	-0.05556
A2M-AS1	-0.195633
A2ML1	0.00945421
A3GALT2	-0.308076
A4GALT	0.129194
A4GNT	0.000072
AAAS	-0.0513608
AACS	-0.114603
AACSP1	1.20951
AADAC	-0.324781
AADACL2	0.00329145
AADACL3	-1.24704
AADACL4	-0.148164
AADAT	0.167376
AAED1	0.253916
AAGAB	0.296782
AAK1	-0.295872

© Copyright Monday, Aug 1, 2016, 3:21 PM by COMPANYNAME

AAMDC	-0.115851
AAMP	-0.260229
AANAT	0.0976664
AAR2	-0.193539
...	...

We will divide the list into 2 sets - one each for up and down regulated genes and then sort the lists individually based on absolute fold-change

Set of Up-Regulated genes

Gene Name	Expression Value
POM121L1P	4.86803
PLAT	4.66066
UGT2B15	4.35543
EGR3	4.33761
PLAC1	4.26765
MYH7	4.23332
MGAT3	4.16032
UGT2B11	4.06999
ARHGAP36	4.04548
AREG	3.97439
RGS22	3.94511
MSC	3.72215
CDH26	3.69005
MAT1A	3.59579
GPR65	3.49472
CALHM3	3.48666
SNCAIP	3.42972
TMPRSS11BNL	3.4246
MGP	3.4027
MYBL1	3.32068
...	...

Set of Down-Regulated genes

© Copyright Monday, Aug 1, 2016, 3:21 PM by COMPANYNAME

Gene Name	Expression Value
HOPX	-5.0717
FAM83C	-4.01407
OR8K5	-3.77881
UPK1A	-3.54601
IL1R1	-3.53915
CLEC17A	-3.41847
HTR1F	-3.40689
LOC285181	-3.3989
C2orf54	-3.38251
SPRY1	-3.3545
LINC00483	-3.28418
MAGEB10	-3.17518
PPEF1	-3.10456
LMO3	-3.08924
PSCA	-3.06494
LOC100129312	-2.96857
FOXB2	-2.94164
WDR49	-2.88707
NEK10	-2.87162
UPK1A-AS1	-2.85906

Step 2 - Calculate enrichment probability of a given ontology category (In this example ERα) using fisher exact probability for top N genes in the list (all up-regulated genes sorted by Fold Change)

Fisher exact test uses the following table to calculate enrichment

Gene List/ Category	Number in gene list	Number not in gene list
**Number in ontology category**	a	b
**Number not in ontology category**	c	d

Where a, b, c and d are calculated as follows

```
a = len(gene_list[0:N].intersection(ESR1_list))
b = len(gene_list[N+1:].intersection(ESR1_list))
```


© Copyright Monday, Aug 1, 2016, 3:21 PM by COMPANYNAME

```
c = len(gene_list[0:N].difference(ESR1_list))
d = len(gene_list[N+1:].difference(ESR1_list))
```

where N = number of genes to be used for enrichment calculation. The results of this calculation is p-values of enrichment

Eg: If N= 10 then top 10 genes i.e from *POM121L1P* to *AREG* from the list above are considered for enrichment calculation above

Step 3 - Repeat Step 2 for N = 1 to len(gene_list) and find peak enrichement

The above process is repeated for multiple slices of the original list. We get a p-value corresponding to each value of N. The following graph shows the plot of -log(p-value) vs N for multiple values of N. Peak enrichment in this case happens at 241 genes(Np) i.e. the top 241 genes contain most information about ERα regulated activity

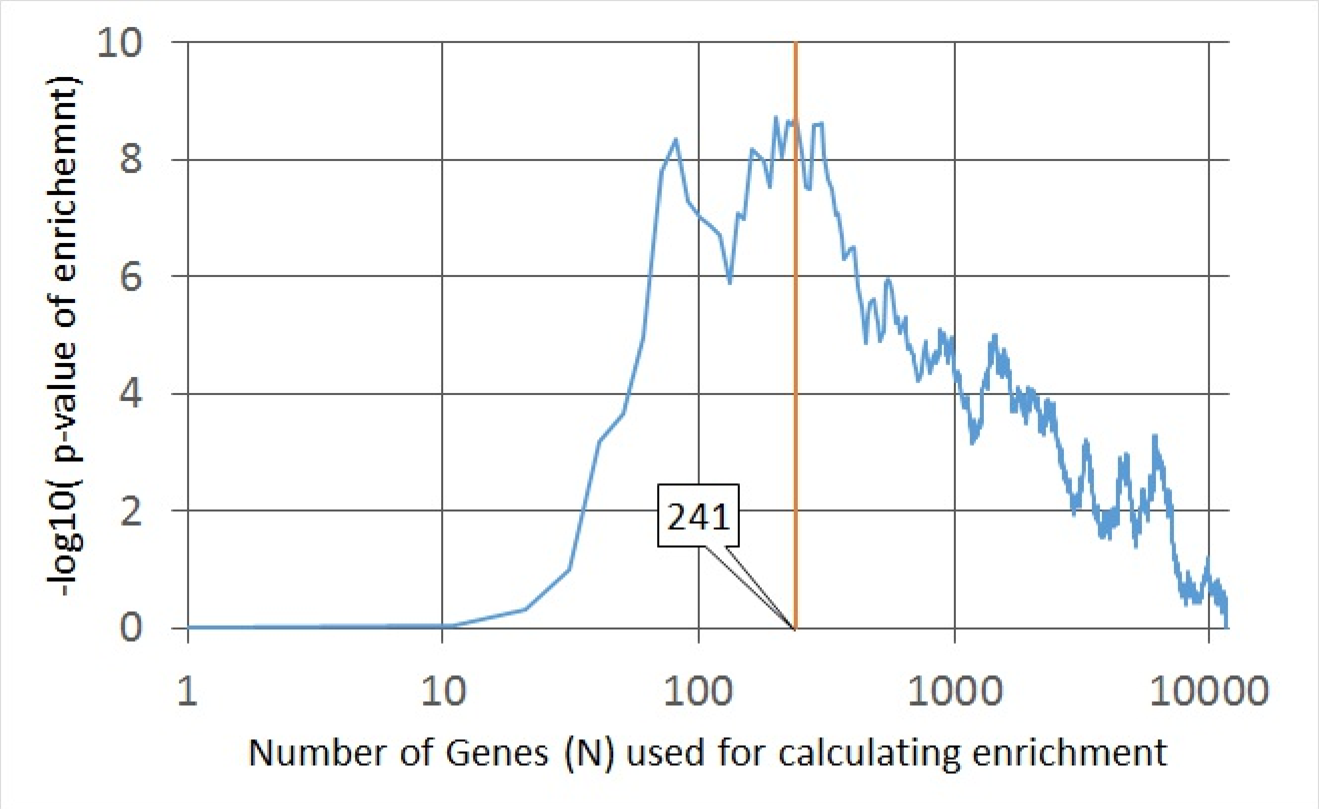

Step 4 - Randomize the gene list selected and repeat step 2 and step 3

Again taking the list of all up-regulated genes, we randomize it such that the genes are no longer sorted based on Fold Change. We then repeat Steps 2 and 3 for each such randomized list created. The following graph shows the plot of -log(p-value) vs N for 100 such randomized calculations.

© Copyright Monday, Aug 1, 2016, 3:21 PM by COMPANYNAME

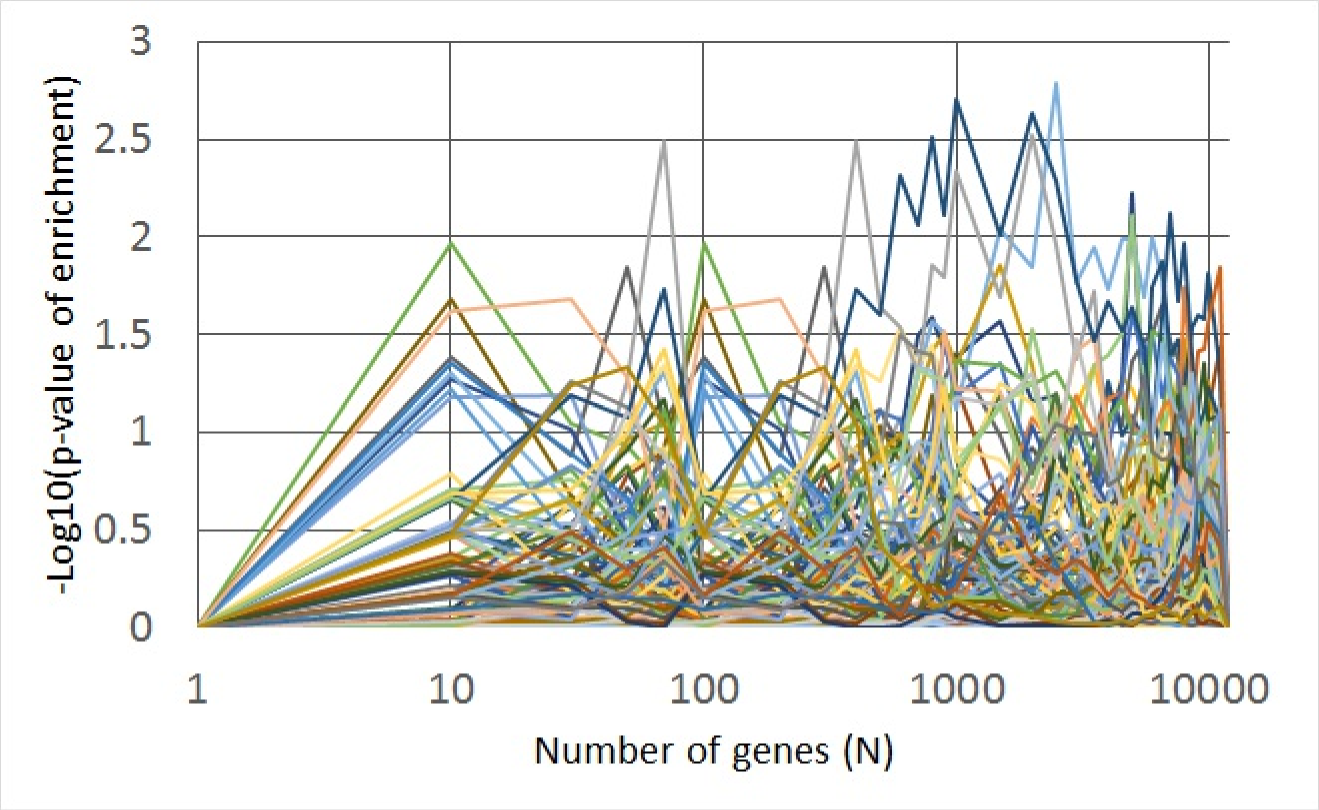

Similar to the the graph above, we select peak enrichment for each iteration. This gives us the background enrichment distribution against which to test the peak enrichment observed in Step 3.

Step 5 - Calculate enrichment e-value for the category.

We finally calculate an e-value for the category by performing a t-test for the p-value of peak enrichment observed in Step 3 against the distribution of p-values at peak enrichment calculated in Step 4

Step 6 - Repeat for each category and perform FDR correction

Steps 2 through 5 are repeated for to get an e-value of enrichment and Np for each category in the ontology. The e-values are then correct for FDR using Benchamini-Hochberg multiple test correction procedure. The e-values and Np obtained for each category are used to interpret the cellular response to treatment.

© Copyright Monday, Aug 1, 2016, 3:21 PM by COMPANYNAME
